# Impact on cardiovascular outcome of coronary revascularization-induced changes in ischemic perfusion defect and myocardial flow reserve

**DOI:** 10.1007/s00259-023-06588-4

**Published:** 2024-01-09

**Authors:** Roberta Assante, Emilia Zampella, Adriana D’Antonio, Teresa Mannarino, Valeria Gaudieri, Carmela Nappi, Parthiban Arumugam, Mariarosaria Panico, Pietro Buongiorno, Mario Petretta, Alberto Cuocolo, Wanda Acampa

**Affiliations:** 1 Department of Advanced Biomedical Sciences, University of Naples Federico II, Via Sergio Pansini 5, 80131 Naples, Italy; 2 Department of Nuclear Medicine, Central Manchester Foundation Trust, Manchester, UK; 3 Institute of Biostructures and Bioimaging, CNR, Naples, Italy; 4 IRCCS Synlab SDN, Naples, Italy

**Keywords:** PET, MPI, Revascularization, Prognosis

## Abstract

**Purpose:**

We evaluated the impact on cardiovascular outcome of coronary revascularization-induced changes in ischemic total perfusion defect (ITPD) and myocardial flow reserve (MFR) as assessed by ^82^Rb positron emission tomography (PET)/computed tomography (CT) imaging.

**Methods:**

The study included 102 patients referred to ^82^Rb PET/CT myocardial perfusion imaging before and after coronary revascularization. All patients were followed for the occurrence of cardiovascular events (cardiac death, nonfatal myocardial infarction, repeated revascularization, and heart failure) after the second imaging study.

**Results:**

During a median follow-up of 20 months, 21 events occurred. The clinical characteristics were comparable between patients with and without events. In the overall study population, after revascularization, there was a significant reduction (*P* < 0.001) of ITPD, while hyperemic myocardial blood flow (MBF) *(P* < 0.01) and MFR (*P* < 0.05) significantly improved. Event rate was higher in patients with ITPD (*P* < 0.005) or MFR (*P* < 0.001) worsening compared to those with unchanged or improved ITPD or MFR. At Cox univariable analysis, ITPD and MFR worsening resulted in predictors of events (both *P* < 0.05). Patients with worsening of both ITPD and MFR had the worst event-free survival (log-rank 32.9, *P* for trend < 0.001).

**Conclusions:**

In patients with stable CAD, worsening of ITPD and MFR after revascularization procedures is associated with higher risk of cardiovascular events. Follow-up MPI with ^82^Rb PET/CT may improve risk stratification in patients submitted to coronary revascularization.

## Introduction

Cardiac imaging by positron emission tomography (PET)/computed tomography (CT) represents a useful noninvasive method for the evaluation of myocardial perfusion and coronary vascular function [1]. The quantification of PET/CT data provides an integrated measurement of myocardial blood flow (MBF), myocardial flow reserve (MFR), and coronary artery calcium content. Therefore, PET/CT is considered a powerful tool in advance current cardiovascular practice in guiding revascularization decisions, potentially for optimal outcomes [2]. The recent European Society of Cardiology guidelines outlined that coronary revascularization has the primary role to improve myocardial perfusion with reducing ischemia [3]. The benefit of coronary revascularization is closely related to the extent of ischemia reduction [4, 5]; however, the clinical impact of revascularization in patient with stable coronary artery disease (CAD) remains to be fully addressed. It has been demonstrated that reduced MFR by PET myocardial perfusion imaging (MPI) is associated with adverse cardiovascular events and patients with low MFR appeared to benefit most from coronary revascularization [6]. However, few studies evaluated the impact on cardiovascular outcome of the changes in myocardial perfusion and absolute MBF after revascularization procedures [7, 8]. Recently, it has been showed that hyperemic MBF and MFR by serial [^15^O]H2O PET may identify patients in whom revascularization will restore myocardial perfusion, potentially improving prognosis [9, 10]. Moreover, ^82^Rb PET demonstrated good repeatability in the serial evaluation of rest and hyperemic MBF measurements [11], supporting an optimal use in quantifying the possible effects of therapeutic interventions on both myocardial ischemia and MBF. The aim of this study was to evaluate the impact on cardiovascular outcome of coronary revascularization-induced changes in ischemic total perfusion defect (ITPD) and MFR as assessed by ^82^Rb PET imaging.

## Methods

### Patients

This retrospective study included 120 consecutive patients with suspected or known CAD who underwent stress-rest ^82^Rb PET/CT at baseline (MPI-1) and at follow-up (MPI-2), after clinically driven coronary revascularization (percutaneous coronary intervention (PCI) or coronary artery bypass grafting (CABG)). The clinical indication for revascularization was performed by referring physician based on angina symptoms, evaluation of stress-induced ischemia, viability testing when available, and invasive coronary angiography. Patients were referred for MPI after revascularization if they were symptomatic. Asymptomatic patients were referred due to incomplete or suboptimal revascularization or as part of risk stratification [12]. The mean interval time between revascularization and MPI-2 was 15 ± 9 months. No cardiac events occurred between coronary revascularization and MPI-2. From the initial cohort of 120 patients, those with previous CABG, left ventricular ejection fraction < 40%, or clinical diagnosis of heart failure before MPI-1 were excluded, leaving a final cohort of 102 patients. As part of the baseline examination, clinical teams collected information on traditional cardiovascular risk factors (including age, sex, body mass index, diabetes, dyslipidemia, smoking, hypertension, and family history of CAD). Patients were classified as having diabetes if they were receiving treatment with oral hypoglycemic drugs or insulin. Hypertension was defined as a blood pressure ≥ 140/90 mm Hg or the use of anti-hypertensive medication [13]. Hypercholesterolemia was defined as total cholesterol level ≥ 6.2 mmol/L or treatment with cholesterol lowering medication. A positive family history of CAD was defined by the presence of disease in first-degree relatives younger than 55 years in men or 65 years in women. Angina symptoms were defined in the presence of typical angina, atypical angina, or non-anginal chest pain [3]. The review committee of our institution approved the study, and all patients gave informed consent (Ethics Committee, University Federico II, protocol number 110/17).

### PET imaging

As a routine preparation for ^82^Rb cardiac PET/CT, patients were asked to discontinue taking nitrates for 6 h, calcium channel blockers and caffeine-containing beverages for 24 h, and beta-blockers for 48 h before their appointment. Scans were acquired using a Biograph mCT 64-slice scanner (Siemens Healthcare). For both rest and stress images, 1110 MBq of ^82^Rb was injected intravenously, and a 6-min list-mode PET study was acquired. Pharmacologic stress was then administered using adenosine (140 μg × kg^−1^ × min^−1^ for 4.5 min). Both rest and stress dynamic images were reconstructed into 26 time frames (12 × 5 s, 6 × 10 s, 4 × 20 s, and 4 × 40 s; total, 6 min) using the vendor standard ordered subset expectation maximization 3D reconstruction (2 iterations, 24 subsets) with 6.5-mm Gaussian post-processing filter. The images were corrected for attenuation using the low-dose CT. Hemodynamic parameters and 12-lead ECG were recorded at baseline and throughout the infusion of adenosine. Trans-axial PET perfusion images were automatically reoriented into short-axis and vertical and horizontal long-axis slices. Regional myocardial perfusion was visually assessed, using standardized segmentation of 17 myocardial regions [14]. Total perfusion defect (TPD) reflecting a combination of both severity and extent of myocardial defect was calculated using automated software (Cedars-Sinai Medical Center, Los Angeles, CA) [15]. The ischemic TPD (ITPD) was defined as stress TPD − rest TPD and expressed as % of left ventricle. A change of ≥ 5% was used as the criterion for a significant serial change in ITPD in an individual patient [16]. The variations in perfusion pattern between MPI-1 and MPI-2 were categorized as improvement when there was a decrease in ITPD value ≥ 5%; no change; and worsening with an increase of ≥ 5% in ITPD.

Absolute MBF (in ml/min/g) was computed from the dynamic rest and stress imaging series with commercially available software (FlowQuant, University of Ottawa Heart Institute) [17]. MFR was defined as the ratio of hyperemic to baseline MBF and was considered reduced when < 2 [18]. Variations in quantitative pattern were categorized as improvement with recovery of MFR form reduced values to normal values after revascularization; no change; and worsening with change in MFR from normal values to reduced values after revascularization.

### Follow-up data

Patient follow-up was prospectively obtained by the use of a questionnaire that was assessed by a phone call to all patients and general practitioners or cardiologists and by review of hospital or physicians’ records by individuals blinded to the patient’s test results. For the purpose of the present investigation, we performed a landmark analysis starting follow-up from MPI-2 [19]. The outcome was a composite endpoint of cardiovascular events including cardiac death, nonfatal myocardial infarction, repeated revascularization, and heart failure, whichever came first. The cause of death was confirmed by review of death certificate, hospital chart, or physician’s records. Death was considered of cardiac origin if the primary cause was defined as acute myocardial infarction, congestive heart failure, valvular heart disease, sudden cardiac death, or cardiac interventional/ surgical procedure related. Myocardial infarction was defined when > 2 of the following 3 criteria were met: chest pain or equivalent symptom complex, positive cardiac biomarkers, or typical electrocardiographic changes [20]. The date of the last examination or consultation was used to determine the length of follow-up.

### Statistical analysis

Continuous data are expressed as mean ± standard deviation and categorical data as percentage. A student two-sample *t*-test and chi-square test were used to compare the differences in continuous and categorical variables, respectively. A *P* < 0.05 (two-sided) was considered statistically significant. Annualized event rates (AER), expressed as % person-years, were calculated as the cumulative number of events divided by person-time. This latter is an estimate of the actual time at risk that all persons contribute to the study, i.e., the sum of each individual follow-up period. Hazard ratios with 95% confidence intervals (CI) were calculated by univariable Cox regression analysis. The incremental prognostic value of clinical data and imaging findings considering variables in hierarchical order was assessed by the likelihood ratio χ^2^. Event-free survival curves were obtained by the Kaplan–Meier method and compared with the log-rank test. Statistical analysis was performed with Stata 12 software (StataCorp, College Station, TX, USA).

## Results

No patients were lost at follow-up. During a median follow-up of 20 months from MPI-2 (range 7–67 months), 21 events occurred (21% cumulative event rate, annual event rate 9.8% person-years). The events were cardiac death in 2 (10%) patients, nonfatal myocardial infarction in 4 (16%), repeated revascularization in 14 (68%), and heart failure in 1 (5%) patient. Baseline clinical characteristics were comparable between patients with and without events (Table 1).

**Table 1:** Baseline clinical characteristics at MPI-1 in patients with and without events

	All patients (*n* = 102)	Event (*n* = 21)	No event (*n* = 81)		*P* value
Age (years)	59 ± 14	63 ± 9		60 ± 9	0.10
Male gender, *n* (%)	76 (73)	14 (66)		62 (82)	0.35
Body mass index (kg/m^2^)	28 ± 5	31 ± 5		28 ± 4	< 0.01
Diabetes, *n* (%)	43 (41)	10 (50)		33 (28)	0.57
Hypertension, *n* (%)	96 (93)	21 (91)		75 (97)	0.19
Dyslipidemia, *n* (%)	89 (88)	21 (87)		68 (90)	0.05
Smoking history, *n* (%)	36 (36)	11 (33)		25 (41)	0.06
Family history of CAD, *n* (%)	49 (47)	13 (44)		36 (51)	0.15
Angina symptom, *n* (%)	66 (67)	16 (61)		50 (74)	0.21
Prior myocardial infarction,* n* (%)	56 (52)	15 (44)		41 (61)	0.08
Prior PCI, *n (%)*	63 (61)	16 (61)		47 (61)	0.14

Values are expressed as mean value ± standard deviation or number (percentage) of patients. *CAD* coronary artery disease, *PCI* percutaneous coronary intervention

**Fig. 1:**
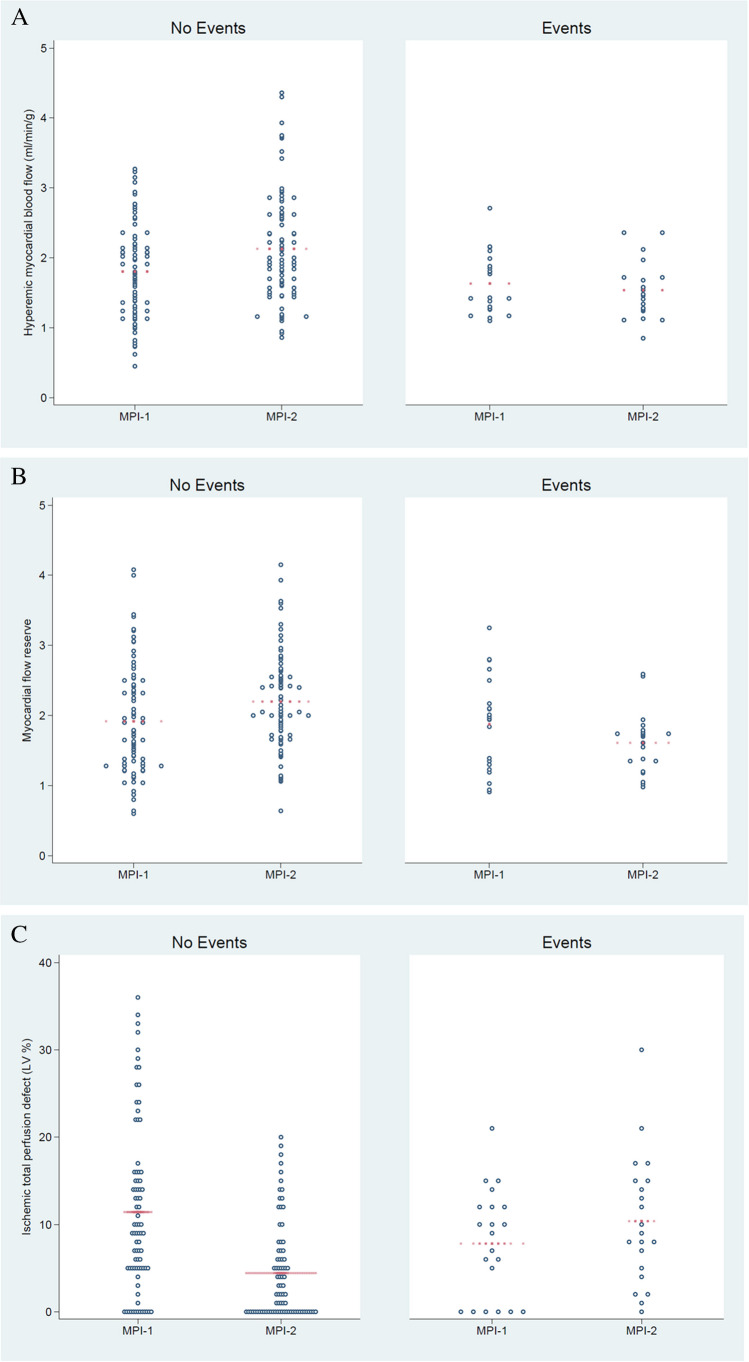
Scatterplots of hyperemic myocardial blood flow (**A**), myocardial flow reserve (**B**), and ischemic total perfusion defect (**C**) at MPI-1 and MPI-2 in patients with and without events. The red dots indicate the mean values

Imaging findings at MPI-1 and MPI-2 in the overall patient population are reported in Table 2. The scatterplots of MPI-1 and MPI-2 perfusion findings in patients with and without events are reported in Fig. 1. Mean values of hyperemic MBF, MFR, and ITPD were comparable between MPI-1 and MPI-2 in patients with events, while MBF and MFR were higher and ITPD lower on MPI-2 compared with MPI-1 in patients without events (Table 3).

**Table 2:** Imaging findings at MPI-1 and MPI-2 in the overall patient population

	MPI-1	MPI-2	*P* value
Baseline MBF (ml/min/g)	0.99 ± 0.33	1.03 ± 0.32	0.41
Hyperemic MBF (ml/min/g)	1.77 ± 0.65*	2.00 ± 0.76*	< 0.01
MFR	1.90 ± 0.75	2.07 ± 0.69	< 0.05
ITPD (%)	10 ± 9	5 ± 7	< 0.001
Scar	5 ± 10	6 ± 10	0.12

Values are expressed as mean value ± standard deviation. *MBF* myocardial blood flow, *MFR* myocardial flow reserve, *ITPD* ischemic total perfusion defect

^*^ *P* < 0.001 vs. baseline

**Table 3:** Imaging findings at MPI-1 and MPI-2 in patients with and without events

	Patients with event (*n* = 21)			Patients with no event (*n* = 81)		
	MPI-1	MPI-2	*P* value	MPI-1	MPI-2	*P* value
Hyperemic MBF (ml/min/g)	1.6 ± 0.4	1.5 ± 0.4	0.47	1.8 ± 0.7	2.1 ± 0.8	< 0.01
MFR	1.8 ± 0.6	1.6 ± 0.4	0.13	1.9 ± 0.7	2.1 ± 0.7	< 0.01
ITPD (%)	7.8 ± 6.1	10.9 ± 9.2	0.20	11.4 ± 9.5	4.4 ± 5.4	< 0.001

Values are expressed as mean value ± standard deviation. *MBF* myocardial blood flow, *MFR* myocardial flow reserve, *ITPD* ischemic total perfusion defect

### Changes in perfusion findings according to events

ITPD improved in 43 (42%) patients, remained unchanged in 45 (44%), and worsened in 14 (14%) patients. MFR improved in 26 (25%) patients, remained unchanged in 60 (59%), and worsened in 16 (16%) patients. At logistic regression analysis, ITPD and MFR at MPI-1 resulted as predictors of ITPD and MFR improvement, respectively (both *P* < 0.01).

Cardiac events were 3 in patients with improved ITPD, 9 in patients with unchanged, and 9 in those with worsened ITPD. The AER was higher (40%) in patients with ITPD worsening compared to those with unchanged (9%) or improved ITPD (3%) (*P* for trend < 0.005).

Patients with improved MFR had no events. Cardiac events were 11 in patients with unchanged and 10 in those with worsened MFR. Accordingly, the AER was higher in patients with worsened MFR (34%) compared to those with unchanged (8%) or improved MFR (0%) (*P* for trend < 0.001).

### Predictors of events

**Fig. 2:**
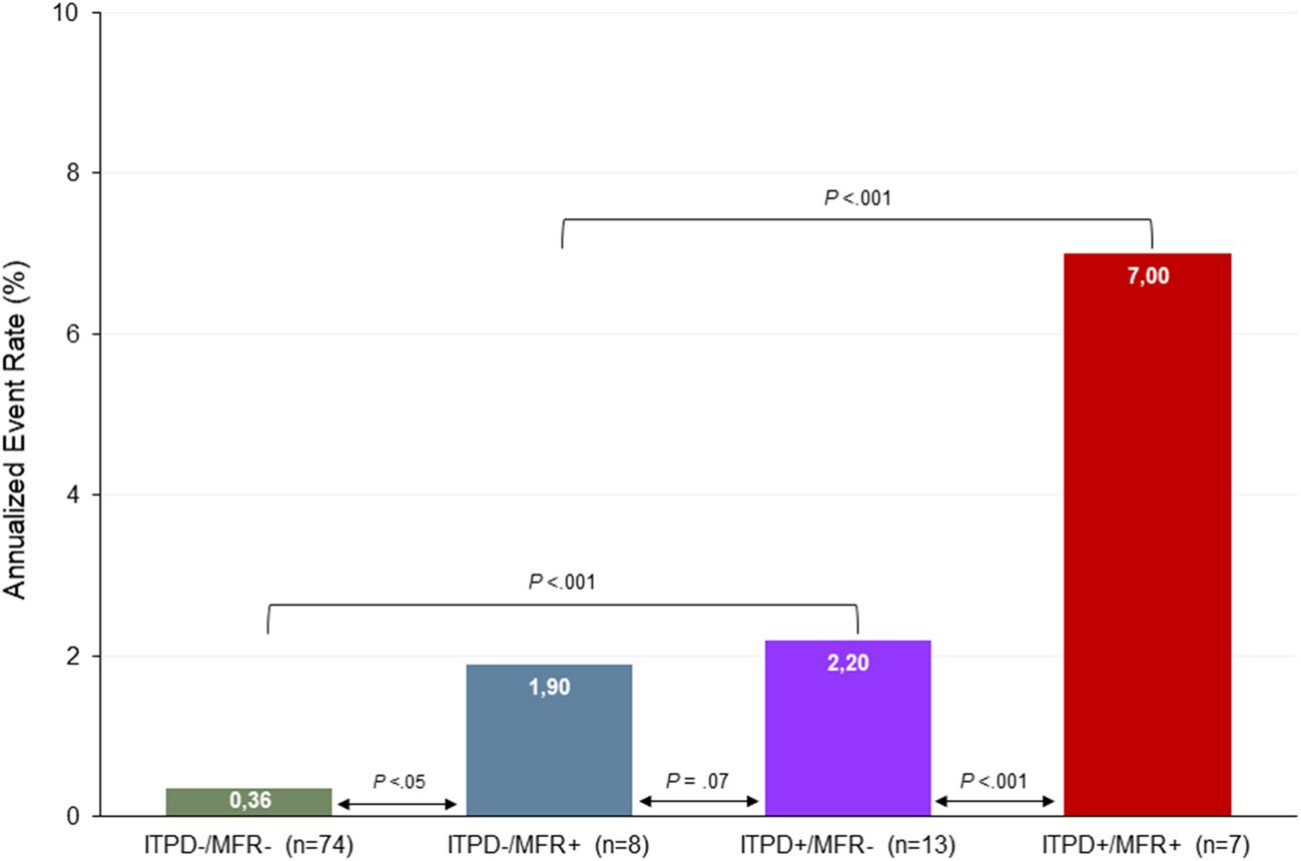
Annualized event rate combining the presence or absence of both ischemic total perfusion defect (ITPD) and myocardial flow reserve (MFR) worsening

**Fig. 3:**
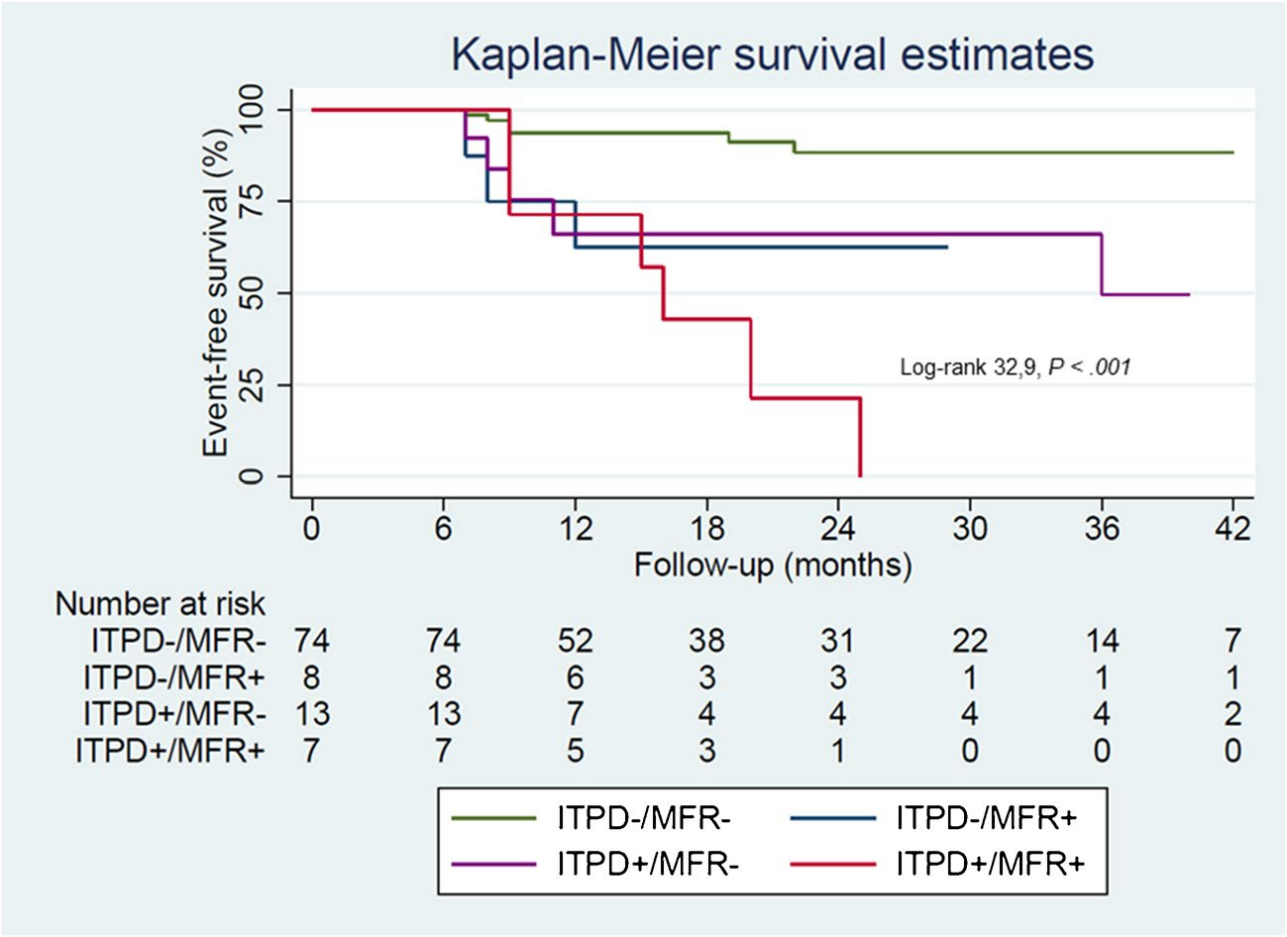
Kaplan–Meier event-free survival curves combining the presence or absence of ITPD and MFR worsening

**Fig. 4:**
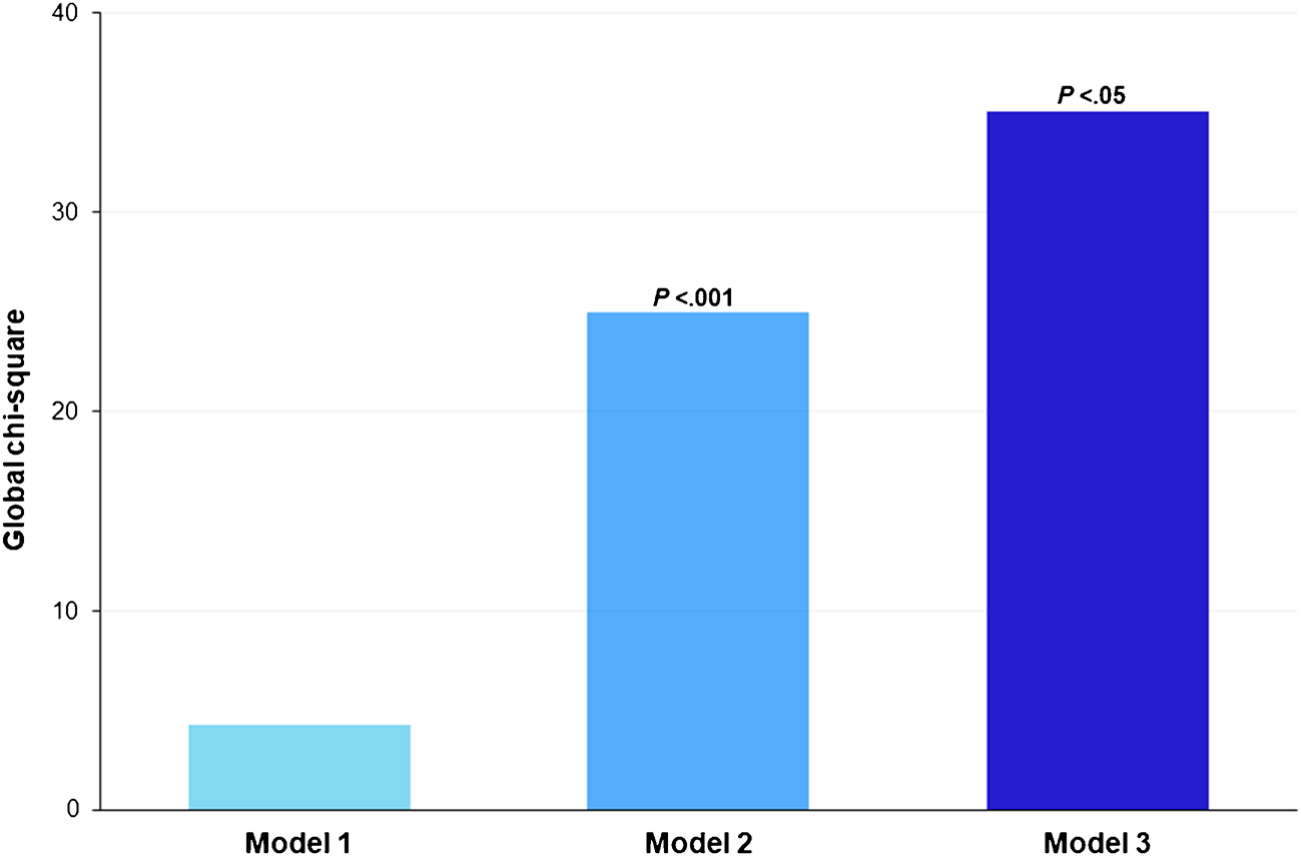
Incremental prognostic value (global χ^2^ values on *y*-axis) of clinical data, ischemic total perfusion defect (ITPD), and myocardial flow reserve (MFR) results added sequentially; model 1, clinical data; model 2, clinical data and ITPD; model 3, clinical data, ITPD, and MFR

At Cox univariable analysis both ITPD and MFR worsening resulted as predictors of events (Table 4). The AER of patients with or without ITPD and MFR worsening is reported in Fig. 2. As showed, patients with ITPD worsening showed a better outcome in the presence of improved or no change MFR compared to patients with both ITPD and MFR worsening (*P* < 0.001). Patients with improvement or no change of ITPD showed a worst outcome in the presence of MFR worsening (*P* < 0.05). The worst outcome was observed in patients with ITPD and MFR worsening (log-rank 32.9, *P* for trend < 0.001) (Fig. 3). At incremental analysis (Fig. 4), the addition of ITPD worsening to a model including only clinical data increased the global χ^2^ from 4.26 to 24.92 (*P* < 0.001). The addition of MFR worsening to a model including clinical data and ITPD further increased the χ^2^ to 35.06 (*P* < 0.05).

**Table 4:** Univariable Cox analysis for cardiac events

	Hazard ratio (95% CI)	*P* value
ITPD change categories		
Unchanged (reference)	–	–
Improved	0.340 (0.092–1.262)	0.10
Worsened	4.183 (1.593–10.985)	< 0.05
MFR changes categories		
Unchanged (reference)	–	–
Improved	0.208 (0.027–1.627)	0.13
Worsened	4.076 (1.686–9.858)	< 0.005

*CI* confidence interval, *MFR* myocardial flow reserve, *ITPD* ischemic total perfusion defect

## Discussion

The present study evaluated the impact of change in myocardial perfusion, MBF, and MFR after coronary revascularization in patients undergoing MPI by ^82^Rb PET/CT. We found that the combined worsening of MFR and ITPD after revascularization was the strongest predictor of poor outcome, associated with a higher risk of events during follow-up.

The quantification of absolute MBF and MFR by PET as markers of coronary vascular function provides an incremental value compared to clinical and perfusion data for both diagnostic and prognostic purposes in patients with suspected or known CAD [21–23]. Previous studies pointed up on prognostic value of MFR [24–27], demonstrating that the coronary vasodilator dysfunction is a powerful and independent correlate of cardiac mortality and provides significant incremental risk stratification [28–30]. The presence of reduced cardiac vascular function was associated with an increased risk of cardiac death even in the presence of normal scans by semi-quantitative visual analysis and independently of other risk factors [31–34]. Myocardial perfusion evaluated by both single-photon emission computed tomography [SPECT] and PET imaging which has been widely used in the follow-up period after revascularization procedure, aiming for guiding the clinical decision-making process and/or to determine the time to retest [34]. It has demonstrated that clinical variables and the presence of myocardial ischemia in post-revascularization patients are useful to predict cardiac events during long-term follow-up [34, 35]. Moreover, both the degree of stress-induced ischemia and left ventricular function can predict the effect of revascularization on outcome in patients with suspected or known CAD [36].

The availability of software for automated reproducible assessment of MPI makes effective the quantitative evaluation of perfusion parameters in serial evaluation [37, 38]. However, few data tested the impact of myocardial perfusion changes in patients on treatment for stable CAD. Moreover, only few data evaluated the impact of changes of myocardial perfusion after revascularization treatment at follow-up.

The prognostic significance of ischemia reduction was examined in a small subgroup of patients, in the adenosine sestamibi SPECT post-infarction evaluation (INSPIRE) study [39]. Ischemia reduction resulted univariate predictor of major adverse cardiac events [40]. In a larger report series, Farzaneh-Far et al. [40] found that 5% worsening ischemia, after medical or revascularization therapy, was a strong predictor of death or myocardial infarction.

As well, the prognostic value of MFR changes specifically after invasive treatment has been poorly investigated. Although the benefit of coronary revascularization is closely related on the severity of baseline myocardial perfusion defects and the extent of ischemia reduction, it has been recently demonstrated that the coronary flow capacity, which combines hyperemic MBF and MFR using [^15^O]H_2_O PET perfusion imaging, represents a diagnostic tool associated with outcome after revascularization therapy [10]. In particular, in patients with a significant amount of ischemic myocardium, the recovery of vasodilator capacity could help to identify those that will benefit most from revascularization. On the contrary, patients in whom the revascularization procedure is not accompanied by an increase in MBF may have a poor outcome.

In our study, all patients underwent ^82^Rb PET/CT for the evaluation of cardiac vascular function and myocardial perfusion. Limited data are available on the value of ^82^Rb PET/CT before and after revascularization procedure, despite MBF by ^82^Rb demonstrated high reproducibility using a same day short-term repeatability protocol [11]. The reproducibility of ^82^Rb MBF assessment is important for serial PET measurements after various therapeutic strategies [11]. In addition, cardiac imaging with ^82^Rb PET/CT is able to provide an accurate measurement of atherosclerotic burden, myocardial perfusion, and coronary vascular function in one examination [41], with a better risk stratification of patient with CAD and prediction of the presence of obstructive CAD [42, 43].

In agreement with a prior study [9], we found that in patients with stable CAD, coronary revascularization was associated with an improvement of hyperemic MBF, MFR, and ITPD. In particular, when we classified patients according to the occurrence of events, those without events showed both a reduction of myocardial ischemic burden and an improvement of coronary blood flow, not detectable in the group of patients with events.

We also found that worsening of ischemic burden as well as of MFR resulted in predictors of cardiac events during the follow-up after coronary revascularization. Not surprisingly, the worst outcome was present in patients with worsening of both parameters. Patients with ITPD worsening showed a better outcome in the presence of an improved or no change MFR compared to patients with both ITPD and MFR worsening. Moreover, patients with improvement or no change of ITPD showed a worst outcome in the presence of MFR worsening. In detail, how impaired MFR is associated with increased clinical risk and precisely how it could modify the effect of revascularization cannot be determined from this study, although from our data it seems that MFR change represents a significant prognostic factor. In our study, population revascularization improved measures of myocardial perfusion in 42% of patients. The presence of residual perfusion abnormalities may be due to incompleteness or failed revascularization procedures or to disease progression potentially associated with limited survival due to residual diffuse CAD. These mechanisms most likely represent the basis for failure of randomized trials to improve survival after revascularization in stable CAD [44].

Our preliminary results involved a limited number of patients in a short-term follow-up. Probably an analysis in a larger study population followed for a longer follow-up time could help to better elucidate the prognostic value of changes in cardiac vascular function after revascularization and clarify if the changes of myocardial flow could be independent by the ischemic burden.

## Conclusions

In patients with stable CAD, the presence of worsening of ITPD and MPR after coronary revascularization procedures was associated with a high risk of cardiovascular events during follow-up. Serial MPI imaging with ^82^Rb PET/CT may improve risk stratification in patients undergoing coronary revascularization.
